# Direct and Label-Free Determination of Human Glycated Hemoglobin Levels Using Bacteriorhodopsin as the Biosensor Transducer

**DOI:** 10.3390/s20247274

**Published:** 2020-12-18

**Authors:** Ying-Chin Lin, Ching-Yu Lin, Hsiu-Mei Chen, Li-Pin Kuo, Cheng-En Hsieh, Xiang-He Wang, Chih-Wen Cheng, Chih-Yin Wu, Yi-Su Chen

**Affiliations:** 1 Department of Family Medicine, School of Medicine, College of Medicine, Taipei Medical University, Taipei 110, Taiwan; green1990@tmu.edu.tw; 2 Department of Geriatric Medicine, School of Medicine, College of Medicine, Taipei Medical University, Taipei 110, Taiwan; 3 Department of Family Medicine, Wan Fang Hospital, Taipei Medical University, Taipei 116, Taiwan; m118108004@tmu.edu.tw (C.-Y.W.); 108358@w.tmu.edu.tw (Y.-S.C.); 4 School of Medical Laboratory Science and Biotechnology, College of Medical Science and Technology, Taipei Medical University, Taipei 11031, Taiwan; cylin@tmu.edu.tw; 5 Department of Chemical Engineering, National Taiwan University of Science and Technology, Taipei 10607, Taiwan; s941609@gmail.com (L.-P.K.); q135135xc@gmail.com (C.-E.H.); a9100290202@gmail.com (X.-H.W.); thomas7983798@gmail.com (C.-W.C.)

**Keywords:** bacteriorhodopsin, glycated hemoglobin, photoelectric biosensor, purple membrane

## Abstract

Glycated hemoglobin (HbA1c) levels are an important index for the diagnosis and long-term control of diabetes. This study is the first to use a direct and label-free photoelectric biosensor to determine HbA1c using bacteriorhodopsin-embedded purple membranes (PM) as a transducer. A biotinylated PM (b-PM) coated electrode that is layered with protein A-oriented antibodies against hemoglobin (Hb) readily captures non-glycated Hb (HbA0) and generates less photocurrent. The spectra of bacteriorhodopsin and Hb overlap so the photocurrent is reduced because of the partial absorption of the incident light by the captured Hb molecules. Two HbA0 and HbA1c aptasensors that are prepared by conjugating specific aptamers on b-PM coated electrodes single-step detect HbA0 and HbA1c in 15 min, without cross reactivity, with detection limits of ≤0.1 μg/mL and a dynamic range of 0.1–100 μg/mL. Both aptasensors exhibit high selectivity and long-term stability. For the clinical samples, HbA0 concentrations and HbA1c levels that are measured with aptasensors correlate well with total Hb concentrations and the HbA1c levels that are determined using standard methods (correlation gradient = 0.915 ± 0.004 and 0.981 ± 0.001, respectively). The use of these aptasensors for diabetes care is demonstrated.

## 1. Introduction

Glycated hemoglobin (HbA1c) is a β-*N*-terminally non-enzymatically glucose-modified form of hemoglobin A (HbA), which is the most abundant and common hemoglobin (Hb) in human erythrocytes [1]. Given the average 100–120 day lifespan of erythrocytes, the ratio of the concentrations of HbA1c to total Hb in blood, the HbA1c level (%), reflects the time-averaged blood glucose levels over the previous 2–3 months. The HbA1c level does not fluctuate as much and as rapidly as blood glucose so it is an important alternative index for the diagnosis and long-term control of diabetes [2]. In 2010, the American Diabetes Association first recommended a diagnostic threshold of ≥6.5% HbA1c as one of the criteria for diabetes. Due to the global increase in the prevalence of diabetes, there is a growing demand for simple, yet accurate and reliable HbA1c analyzers, which can be readily operated by both health-care professionals and lay users.

A variety of analytical technologies are used to determine HbA1c levels in human blood, including aptamer assay, boronate affinity chromatography, capillary electrophoresis, enzymatic method, colorimetric method, chemiluminescence, immunoassay, electrochemical and optical biosensors, ion-exchange chromatography, mass spectroscopy, quartz crystal microbalance, spectroscopy, and surface plasmon resonance [3,4,5,6,7,8,9,10,11,12,13,14]. The standard reference method that was developed and approved by the International Federation of Clinical Chemistry (IFCC) for HbA1c assays involves initial proteolytic digestion of total Hb with an endoproteinase, separation by high-performance liquid chromatography and quantification by either mass spectrometry or capillary electrophoresis coupled with ultraviolet detection [11,15]. However, this method requires sophisticated instruments and professional skills, so it cannot be used in clinics or in the home.

Charges, glycans, immunity, structural affinity, and vibrational modes are the principles that are commonly employed to distinguish HbA1c from other Hb molecules [4,7,12,15,16,17,18,19,20]. Affinity-based assays, which simply engage selective antibodies, aptamers, boronates, or molecularly imprinted polymers as the recognition elements to directly capture HbA1c, allow direct, easy, and rapid diagnosis, so they are frequently applied to construct HbA1 biosensors or point-of-care devices [8,12,21]. Recently, nucleic acid aptamers, which are easily synthesized and inexpensive and which exhibit high affinity, selectivity and stability, are used toward Hb and HbA1c and are used in the development of the chemiluminescence-based and electrochemical aptasensors to determine HbA1c levels [6,19,22].

Bacteriorhodopsin (BR), which is a natural photosensor protein that resides in the purple membrane (PM) of *Halobacterium salinarum,* is the photoelectric transducer for novel immunosensors that have recently been developed for direct and label-free detection of microorganisms [23,24]. BR naturally exists as trimers that are arranged in hexagonal lattices of 6.2 ± 0.2 nm side length and which are repeatedly and densely assembled inside a stable 5-nm-thick two-dimensional (2D) PM with a packing density of around 2.6 × 10^4^ trimers/μm^2^, according to the atomic force microscopy images (AFM) [25]. Driven by the photoisomerization of its retinal chromophore, each illuminated BR monomer pumps a proton uni-directionally from the cytoplasmic (CP) to the extracellular (EC) side of PM and produces a photoelectric effect that is applied in bioelectronics devices [26,27]. The photocurrent that is produced by BR correlates well with the incident light intensity [28] so the light-shielding bacterial cells that are captured by antibodies that are attached to PM-coated electrodes are readily detected using the reduction in photocurrent in the immunosensors as a simple quantification parameter [23,24]. Material that binds on the immobilized BR molecules may also cause restricted movement in the helices that are involved in proton transportation and contribute to the reduction in photocurrent generation during the layer-by-layer preparation of the sensor and in target binding [23].

Light-activated BR transports protons uni-directionally, so counter-oriented PM layers generate opposite proton fluxes and thus produce photocurrent ineffectively. To maximize sensor efficiency, the principle of strep(avidin)-biotin affinity adsorption is used to achieve unidirectional immobilization of PM layers on electrodes [23,24]. Moreover, double-sided avidin-linked graphene oxide (GO) sheets and shear flow are innovatively applied to PM fabrication to attain a high-coverage and uniformly oriented PM monolayer to effectively facilitate subsequent conjugation of recognition molecules on top [24].

This study reports the first use of BR-based photoelectric biosensors for direct and single-step assays of non-glycated Hb (HbA0) and HbA1c without labeling. HbA0 and HbA1c are significantly smaller than bacteria so they have very little light-scattering effect, but their absorption spectra overlap with that of bR in the wavelength region of 500–640 nm [13,29,30,31] so it is likely that HbA0 and HbA1c molecules that are captured on PM-coated electrodes hinder movement of the helices of BR for proton pumping and block part of the incident light on BR, so the photocurrent is reduced.

This method of detection is verified using a BR-based Hb immunosensor that is prepared with a versatile protein A-conjugated PM electrode that was developed by the authors [24]. Two other aptasensors are devised by covalently attaching HbA0 and HbA1c-specific nucleic acid aptamers [18] on PM electrodes to, respectively, quantify HbA0 and HbA1c. The selectivity and sensitivity of both aptasensors is studied and they are used to determine HbA1c levels in human whole blood.

## 2. Materials and Methods

### 2.1. Materials

PM was ultracentrifugation-purified from shake-flask cultures of *H. salinarum*, following the standard protocol [29]. In brief, harvested cells were dialyzed against deionized water containing DNase and then centrifuged at 13,000× *g* to collect the purple debris. After washed with 0.1 M NaCl and deionized water several times by centrifugation at 40,000× *g*, the purple pellet was resuspended in deionized water, layered over a 30% to 50% (*w*/*v*) sucrose density gradient with 60% (*w*/*v*) sucrose bottom cushion, and then subjected to ultracentrifugation at 100,000× *g*. The purple band was collected, diluted with deionized water, and washed by repeated centrifugation to remove residual sucrose. Finally, the purple pellet, i.e., PM, was resuspended in deionized water, aliquoted, and stored at −20 °C until use. The purity of PM was confirmed by sodium dodecyl sulphate–polyacrylamide gel electrophoresis and ultraviolet–visible spectroscopy.

b-PM, a biotinylated form of PM, and oxidized avidin (OA) were produced by, respectively, modifying PM and avidin with EZ-Link sulfo-NHS-LC-LC-Biotin and sodium periodate [32]. The HbA0- and HbA1c-specific aptamers were synthesized by Integrated DNA Technologies, with sequential addition of a primary amino group and a dodecane (C12) linker at the 5′ ends of their respective, previously reported sequences [18], (HbA0: 5′-aminoC12-GGC AGG AAG ACA AAC ACC AGG TGA GGG AGA CGA CGC GAG TGT TAG ATG GTA GCT GTT GGT CTG TGG TGC TGT-3′; HbA1c: 5′-aminoC12-GGC AGG AAG ACA AAC ACA TCG TCG CGG CCT TAG GAG GGG CGG ACG GGG GGG GGC GTT GGT CTG TGG TGC TGT-3′). 3-Aminopropylphosphonic acid (APPA) was obtained from Acros. Human HbA1c and recombinant *Staphylococcus aureus* protein A were both obtained from ProSpec. Avidin, bis(sulfosuccinimidyl) suberate (BS3), EZ-Link sulfo-NHS-LC-LC-Biotin, sulfosuccinimidyl(4-iodoacetyl)aminobenzoate (Sulfo-SIAB) were obtained from Thermo Fisher Scientific. Human HbA0 (product no. H0267), poly (ethylene glycol) with a number average molecular weight of 300 (PEG300), rabbit polyclonal antibodies raised against human Hb and all the chemicals that were used in the interference study were obtained from Sigma-Aldrich. Goat anti-Rabbit IgG H&L (Alexa Fluor^®^ 488) was obtained from Abcam. Graphene oxide (GO) powders and indium tin oxide (ITO) glass (sheet resistance: <15 Ω/sq) were, respectively, obtained from Graphene Supermarket and Fang materials, New Taipei City, Taiwan.

### 2.2. Preparation of Sensing Chips

ITO glass electrodes were coated with b-PM monolayers, which are termed b-PM chips, and were prepared as previously described [24]. APPA-aminated ITO glass was drop-coated with a GO-OA complex linker, which was prepared by mixing GO and OA at a 1:10 weight ratio, and then 1.5 mg/mL b-PM (coating diameter: 3 mm) and was subsequently washed with a laminar shear flow (Reynolds number = 0.9) inside a microfluidic setup. The mixing weight ratio of GO and OA was set at 1:10 because we observed a highest photocurrent production by the resulting b-PM chip in another unreported study. To prepare Hb immunosensor, the resulting b-PM chip was sequentially deposited with excess BS3, protein A, glycine and anti-human Hb antibodies, as described in a previous study [24]. To prepare HbA0 and HbA1c aptasensors, the HbA0- and HbA1c-specific aptamers were first modified with excess Sulfo-SIAB, dialyzed against 10 mM phosphate buffer (PB) at pH 8.5 and then drop-coated on the b-PM chip at 2 μM. After a brief rinse, all sensing chips were either directly subject to analysis, or stored in 10 mM PB, containing 100 mM NaCl at pH 8.0 and 4 °C.

### 2.3. Detection of Pure HbA0, HbA1c, and Clinical Samples

Five μL of samples that were suspended in the designated binding buffers were drop-coated onto their respective sensing chips for direct and single-step assay. For the Hb immunosensor assay, 10 mM PB at pH 7.4 was used as the binding buffer and the coating was performed at 4 °C for 2 h. For both HbA0 and HbA1c aptasensor assays, a binding buffer of 150 mM NH_4_Cl, 10 mM NaHCO_3_, 1 mM EDTA-Na_2_, 500 mM NaCl and 50% PEG 300 at pH 7.4 was used and the coating was conducted at room temperature for 15 min. After the coating, the chip was briefly rinsed with a binding buffer and the photocurrent was measured.

Human whole-blood clinical samples were collected from patients in Taipei Medical University-Shuang Ho Hospital (New Taipei City, Taiwan), with the approval by the Taipei Medical University (TMU)-Joint Institutional Review Board (N201806020, 10 July 2018). All volunteers, 14 diabetic (DM) (6 men and 8 female, 56.2 ± 12.5 years old) and 5 non-diabetic (Non-DM) patients (3 men and 2 female, 43.2 ± 10.8 years old) gave informed consent to participate. The corresponding total Hb concentrations and HbA1c levels were determined using a Beckman Coulter DxH 800 hematology analyzer (Miami, FL, USA) and the Sebia CAPILLARYS Hb A1c kit with a Sebia CAPILLARYS 2 FLEX-PIERCING instrument (Paris, France) in the Department of Laboratory Medicine of Taipei Municipal Wanfang Hospital (Taipei, Taiwan). For the photoelectric sensor analysis, the collected whole-blood samples were first, respectively, diluted 5000- and 1000-fold with the binding buffers of the HbA0 and HbA1c aptasensors and then 5 μL of the dilute solutions were subject to the sensor analysis that is described above.

**Figure 1:**
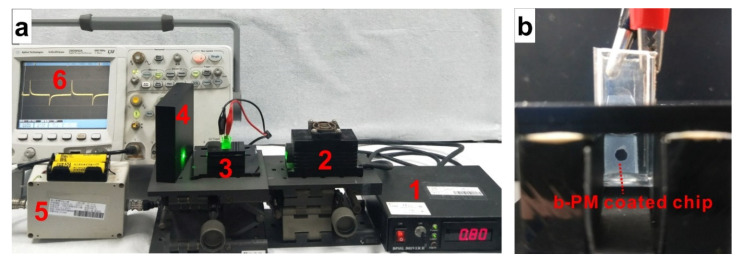
Photos of (**a**) the equipment setup for photocurrent measurement and (**b**) the detection cell. In (**a**), 1: laser power controller; 2: 532 nm CW laser; 3: detection cell and the holder; 4: beam dump; 5: current amplifier; 6: oscilloscope. In (**b**), a b-PM coated chip and a platinum bar are positioned inside a cuvette and then connected to the current amplifier through the black and red wires, respectively. The b-PM chip is prepared with an APPA-aminated ITO glass that is covered with a holed tape, following the procedure described in the Section 2.2. The hole has a diameter of 3 mm, and is made to confine material coating and to ease laser-beam alignment.

Figure 1 shows the setup for photocurrent measurements of b-PM chips. The measurement was performed inside a cuvette, as described previously [24], using an 80-mW green CW laser (beam diameter: 3 mm) as the light source, a platinum bar as the counter electrode and 1 mM PB, 10 mM KCl at pH 8.5 as the electrolyte. The transient photocurrent signals that respond to an on-and-off irradiation cycle that is composed of a 2–3 min continuous illuminations and then a 2–3 min light interruptions were collected in real-time using a homemade current amplifier and were recorded using a digital oscilloscope. The difference in the photocurrent density between the maximum light-on and the minimum light-off signals was used to define the total photocurrent density of the chip. The photocurrent reduction level is defined as the reduction in the total-photocurrent-density percentage of the sensing chip after incubation with the sample solution from the total photocurrent density of another control chip that is incubated with only the blank binding buffer as the reference. The reduction in the photocurrent due to the detection of pure HbA0 and HbA1c solutions (0.1–100 μg/mL) was used to construct the calibration curves for their respective aptasensors and to determine HbA0 and HbA1c concentrations of the diluted clinical samples, [HbA0] and [HbA1c]. The HbA1c level is determined using Equation (1).

(1) $$\mathrm{HbA}1c\;\left(\%\right)=100\cdot \frac{\left[\mathrm{HbA}1c\right]}{\left[\mathrm{HbA}1c\right]+\left[\mathrm{HbA}0\right]}\;$$

### 2.4. Characterization of Sensing Chips

Atomic force microscopy (AFM) was performed on samples that were prepared on freshly cleaved mica, using coating procedures that are exactly the same as those for ITO. The analysis was performed in air and at room temperature, using a Bruker Dimension Icon Scanning Probe Microscope equipped with a Bruker ScanAsyst-Air probe that was operated in PeakForce mode. Raman spectra were collected using a UniNano Tech UniG2D Raman Spectroscope with a 50 mW 532 nm CW laser as the light source.

## 3. Results and Discussions

### 3.1. BR and Hb Absorption Spectra

**Figure 2:**
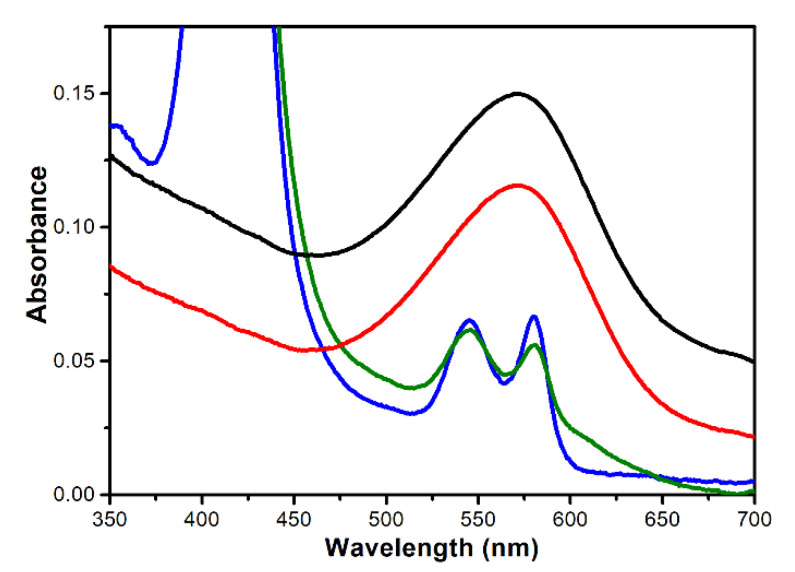
Overlapping absorption spectra of (**red**) 50 μg/mL b-PM, (**black**) 50 μg/mL PM, (**blue**) 100 μg/mL HbA0 and (**green**) 100 μg/mL HbA1c. The monomeric extinction coefficients of b-PM, PM, HbA0 and HbA1c at 532 nm are calculated to be 49,451, 68,061, 7368 and 9066 cm^−1^∙M^−1^, respectively.

Figure 2 shows the overlapping absorption spectra of the chromophoric proteins that are used for this study, each of which are similar to previously reported data [13,29,30,31]. HbA0 and HbA1c have similar spectra with two characteristic peaks at 546 and 580 nm, respectively, so both proteins are mostly in their oxygenated forms [30,33]. The broad spectra of PM and b-PM, a PM conjugated with biotin at its EC side are very similar. Both peak at 572 nm and entirely cover the two peaks of HbA0 and HbA1c. b-PM exhibits lower absorbance than PM due to biotin conjugation. The monomeric extinction coefficients of HbA0 and HbA1c at 532 nm, which is the laser wavelength illuminating the BR-based sensing chip, are 15% and 18% of the value of b-PM, respectively. Therefore, Hb and HbA1c molecules that are adsorbed on top of immobilized b-PM partly absorb incident light irradiating on BR and hence less photocurrent is produced in the sensing chip.

### 3.2. Hb Immunosensor

**Figure 3:**
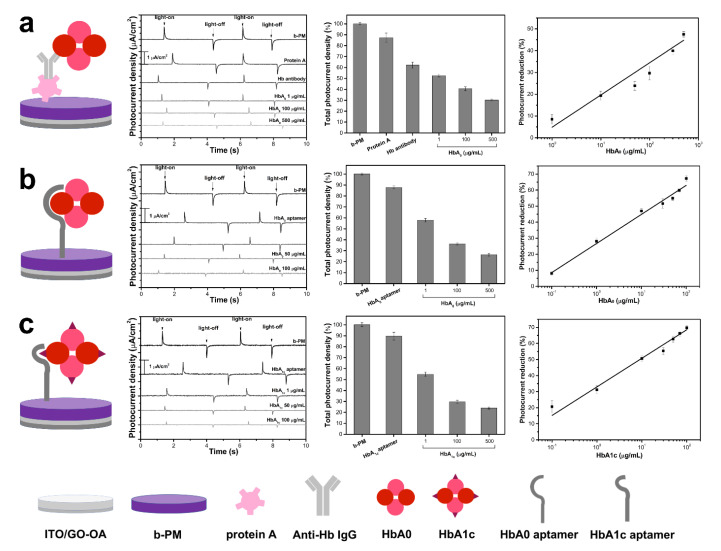
(Left) Structural schemes, (left center) typical photocurrent responses, (right center) total photocurrent density and (right) calibration curves for (**a**) Hb immunosensor, (**b**) HbA0 aptasensor and (**c**) HbA1c aptasensor. The left center and right center panels also show the data for chips that are fabricated using different top layers during the layer-by-layer sensor fabrication. All of the data in the right center and right panels is presented as the average value for three chips of a single type with one standard deviation (RSD < 5%).

A Hb immunosensor was first devised to determine the feasibility of direct Hb detection using b-PM as a transducer. As shown in the left panel of Figure 3a, the sensing chip was prepared by adsorbing Hb-specific antibodies on a protein A-coated b-PM chip, which was constructed by covalently conjugating protein A on a large, nearly continuous and unidirectional b-PM monolayer that was formed on the ITO electrode using a double-sided planar GO-OA complex linker and a post-deposition washing procedure [24]. The planar complex linker had OA molecules conjugated on both basal planes of a GO sheet, so it was horizontally positioned on an APPA-aminated electrode through the Schiff’s base linkage between the terminal amines of APPA and the aldehydes of oxidized sugars on avidin. The planar complex linker also provides a flat support for subsequent directional attachment of b-PM patches through avidin-biotin affinity adsorption. The post-deposition washing was performed to provide a shear flow to mobilize and segregate stacking b-PM patches to achieve high-coverage b-PM monolayer fabrication, as well as effective subsequent protein A conjugation. The photocurrent responses that are generated from each type of b-PM coated electrode are shown in the left center panel of Figure 3a. All resemble the previously reported signal, which comprises a pair of two transient spikes with opposite polarity [23,24]. The right center panel of Figure 3a shows a gradual reduction in the total photocurrent density for different b-PM chips that are successively coated with protein A, blocking glycine, anti-Hb antibodies and HbA0 on top, which confirms that the material binds during the layer-by-layer coating process. The reduction in the photocurrent for the immunosensor rose with increasing HbA0 coating concentrations (Figure 3a, right panel). This suggests that HbA0 binding affects the photocurrent that is generated by the coated BR molecules and this effect increases with the HbA0 binding amount. The reduction in photocurrent may be due to light absorption, as mentioned previously, and the inhibition of BR helix movement due to captured HbA0 molecules. Therefore, the b-PM coated electrode is confirmed to be a suitable transducer to directly quantify Hb.

### 3.3. HbA0 and HbA1c Aptasensors

Antibodies are expensive and unstable, so this study uses a HbA0-specific nucleic acid aptamer [18] as the recognition element to fabricate a sensor. The aptamer was first 5′-modified with a primary amino group to immobilize on top of the pristine b-PM monolayer chip using Sulfo-SIAB as the heterobifunctional crosslinker, which contains an iodoacetyl and a sulfosuccinimidyl group at either end. The sulfosuccinimidyl group was first reacted with the terminal amino group of the aptamer to give an iodoacetyl derivative that readily conjugates to the methionine residues on the exposed CP side of the b-PM chip, according to a previous interpretation [23]. The left panel of Figure 3b shows the structure of the HbA0 aptasensor that is bound with HbA0. The aptamer conjugation and the subsequent HbA0 binding were confirmed by the noticeable successive reduction in photocurrent (Figure 3b, left and right center panels). Similarly to the Hb immunosensor, the reduction in the photocurrent in the aptasensor increases with the HbA0 coating concentrations (Figure 3b, right panel), so quantitative Hb detection is possible with the as-prepared aptasensor. The HbA0 aptasensor has a monomeric dissociation constant (K_d_) of 79 ± 36 nM (Figure S1a), which is 10 times greater than the previously reported value [18]. The difference is attributed to the difference in the assay methods. For this study, the sensing aptamers are immobilized on a solid electrode surface to capture free HbA0 molecules. A magnetic bead-based chemiluminescence assay was employed in a previous study, whereby HbA0 molecules are immobilized on suspended magnetic beads to capture the free sensing aptamers. Since suspended HbA0-coated magnetic beads have a greater mobility than a static aptamer-coated electrode, the chance of interaction between HbA0 and the aptamers is higher in the previous study than in this study. Therefore, a higher affinity and hence a smaller K_d_ value were obtained in the previous study.

**Figure 4:**
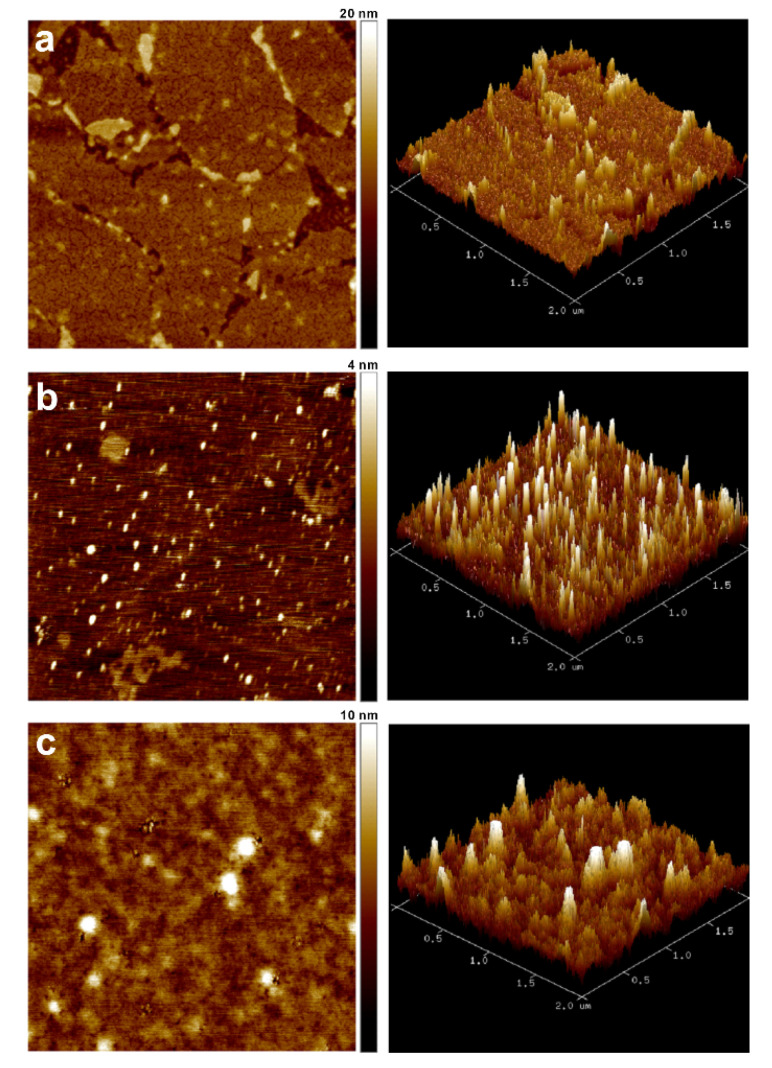
AFM (left) topographic and (right) 3D images of different layer-by-layer fabricated mica. The topmost layer in each image is (**a**) b-PM, (**b**) HbA0 aptamer and (**c**) HbA0: Scan size: 2 μm.

**Figure 5:**
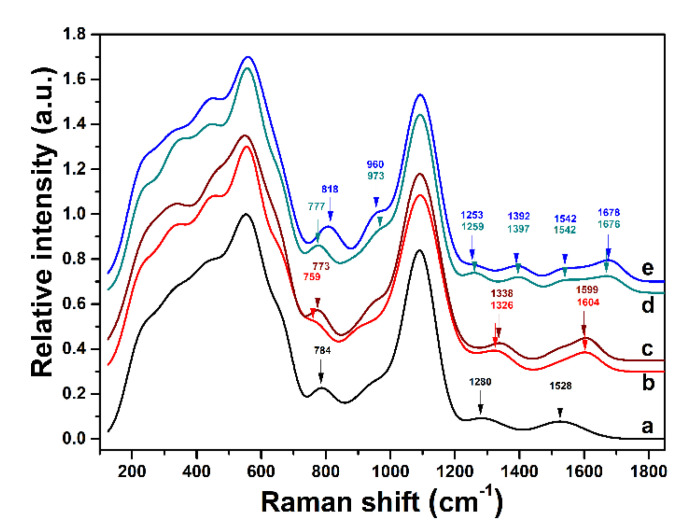
Normalized Raman spectra for ITO electrodes that are fabricated using (**a**) b-PM, (**b**) HbA0 aptamer, (**c**) HbA1c aptamer, (**d**) HbA0, and (**e**) HbA1c at the top. The HbA0 aptamer and HbA1c aptamer are coated on the (**a**) surface to yield surfaces (**b**) and (**c**). Surfaces (**b**) and (**c**) are then used to capture HbA0 and HbA1c on top to yield surfaces (**d**) and (**e**), respectively.

The HbA0 molecules that are captured on the aptasensor were first identified by immunostaining to reveal the green fluorescent surface where the immobilized HbA0 is labeled with anti-human Hb antibodies (Figure S2). AFM (Figure 4) and Raman (Figure 5) analysis were used to confirm the layer-by-layer molecular coating on the pristine b-PM chip to prepare the HbA0-aptasensor and to subsequently capture HbA0. As shown in Figure 4, the topographic AFM images of the topmost b-PM, HbA0 aptamer and of the HbA0 layers on mica are significantly different, which demonstrates successive material coatings. The pristine b-PM layer in Figure 4a resembles a large monolayer and is mainly comprised of contiguous, irregularly shaped and small monolayers with partly connected borders and with a few extra tiny layers sparsely overlaid on top. This pattern is characteristic of mobilization and redistribution of the deposited b-PM patches during the post-deposition washing process, as determined by a previous study by the authors [24]. The cracked morphology in the interior of each b-PM small layer, which is identified as the CP side of PM [34], suggests that all b-PM patches are immobilized with their EC side anchored on the immobilized GO-OA linker. Both HbA0 aptamers and HbA0 molecules are uniformly distributed, but have different surface textures. The nucleic acid aptamers appear as narrow, long rods (Figure 4b) and the HbA0 layer resembles the Chocolate Hills (Figure 4c), each of which match the biological conformations of these two molecules.

The Raman spectra for the pristine b-PM chip, the HbA0 aptasensor and the HbA0-coated HbA0 aptasensor overlap in Figure 5. The bands are identified by de-convolution, as shown in Figure S3, and then tabulated and assigned in Table S1, according to the results of previous studies. The broad band for the b-PM chip at 784 cm^−1^ (Figure 5a) is attributed to Trp residues and the weak bands at 1280 and 1528 cm^−1^ are attributed to the C-C (C=C) stretching and C-C-H in-plane bends of the retina in BR. Further HbA0-aptamer coating is evidenced by the 1326 and 1604 cm^−1^ bands in the spectrum for the HbA0 aptasensor (Figure 5b), which characterizes DNA bases. After the deposition of HbA0, additional Hb characteristic bands are observed at 973, 1259, 1397, 1542 and 1676 cm^−1^ (Figure 5d). The difference in the spectra confirms the different compositions of the successively coated materials on the electrode.

The same fabrication method was used to produce HbA1c aptasensors. The structure of the HbA1c aptasensor that is bound with HbA1c is shown in the left panel of Figure 3c. The immobilization of HbA1c-aptamer on the b-PM chip is demonstrated by the presence of the DNA-base bands at 1338 and 1599 cm^−1^ in the Raman spectrum of the HbA1c aptasensor (Figure 5c). When HbA1c is adsorbed, the spectrum exhibits weak Hb bands at 960, 1253, 1392, 1542 and 1678 cm^−1^ and another strong band at 818 cm^−1^ (Figure 5e), which is the unique characteristic band of HbA1c. The fabrication and the HbA1c binding of the HbA1c aptasensor are confirmed.

Similarly to the HbA0 aptasensor, the coating of the HbA1c aptamers resulted in a significant decrease in the photocurrent for the b-PM chip, as well as the subsequent binding of HbA1c (Figure 3c, left and right center panels). The reduction in the photocurrent for the HbA1c aptasensor also increases with the HbA1c coating concentrations (Figure 3c, right panel), with a K_d_ of 84 ± 43 nM (Figure S1b). Therefore, simultaneous quantification of Hb and HbA1c using their respective aptasensors is feasible. The gradients of the calibration curves for HbA0 and HbA1c aptasensors are similar (18.2 ± 0.8 vs. 17.8 ± 1.1), which demonstrates that both aptasensors have similar detection mechanisms and a similar binding affinity toward their respective targets. At the lowest detection concentration (0.1 μg/mL), the HbA1c aptasensor exhibits a significantly greater decrease in photocurrent than the HbA0 specimen did (20.6 ± 3.7% vs. 8.1 ± 1.2%), which demonstrates a lower detection limit. As shown in Figure 2, HbA1c has a higher molar absorptivity than HbA0, so HbA1c binding results in a greater decrease in photocurrent than HbA0 binding.

The selectivity of the HbA0 an HbA1c aptasensors is determined. As shown in Figure S4, there is no significant decrease in the photocurrent when the HbA0 aptasensor is incubated with HbA1c and vice versa, which demonstrates that both aptasensors exhibit no cross reactivity with each other and that this biosensor system distinguishes HbA1c from HbA0. Several common interferents that may affect the HbA1c assay were also tested, including endogenous substances and exogenous over-the-counter and oral antihyperglycemic drugs [35,36,37]. Table S2 shows that there is no significant interference for all of the tested substances up to the stated concentrations.

**Figure 6:**
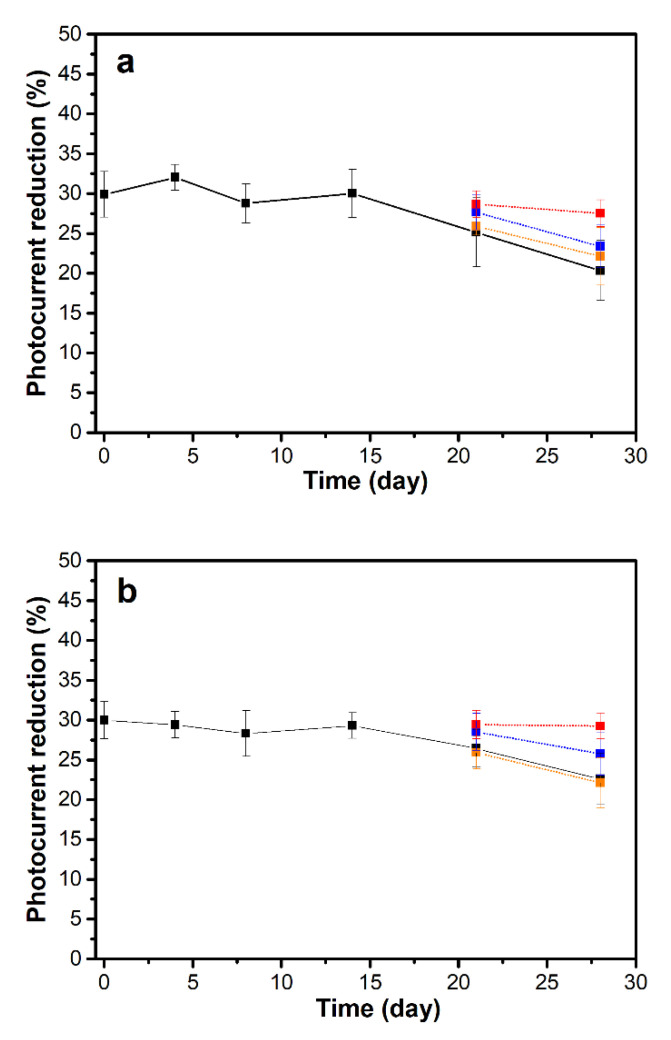
The effect of storage on the decrease in the photocurrent in (**a**) HbA0 and (**b**) HbA1c aptasensors for the detection of 1 μg/mL pure HbA0 and HbA1c, respectively. The aptasensors were stored in 10 mM PB containing 100 mM NaCl at pH 8.0 and 4 °C for 0–28 days. After storage, the aptasensors were either (black) directly used to detect their respective targets or (orange, blue, red) refolded and then used for target detection. The refolding process involved thermal denaturation of the stored aptasensors at 45 °C for (orange) 10 min, (blue) 20 min and (red) 30 min, followed by a 30-min cooling period. All the data is presented as the average value for three chips of a single type with one standard deviation (RSD < 5%).

To determine the stability of the HbA0 an HbA1c aptasensors in storage, the physical stability of the b-PM transducer layer is determined. As shown by the AFM in Figure S5, the b-PM coating layer remains firmly attached to the substrate surface after proper storage for 14 and 28 days. The photoelectric activity of the b-PM chip remains almost the same for the first 21 days and decays only slightly at the 28th day (Figure S6a). Similarly, both HbA0 and HbA1c aptasensors remain fully photo-electrically active at the 28th day (Figure S6b,c). However, the detection ability of these two aptasensors toward their respective targets decays noticeably at the 21st day of storage and further at the 28th day, according to the measured decrease in photocurrent in Figure 6 (black points). This decay is attributed to the formation of incorrect secondary conformations of the immobilized aptamers during prolonged low-temperature (4 °C) storage. To restore the target binding affinity for these misfolded aptamers, a mild refolding process was performed before target binding for aptasensors that were stored for 21 and 28 days. The stored cool aptasensors were first denatured at 45 °C for 10 to 30 min and then refolded at room temperature for another 30 min. Higher denaturation temperatures were not used for this study due to possible deterioration of the layer-by-layer structure and the photoelectric activity of the aptasensors. The results that are shown in Figure 6 (orange, blue and red points) show that both aptasensors gradually recover their target binding ability as the heating time increases. If a 30 min thermal denaturation is employed, for the HbA0 aptasensors that are stored for 21 and 28 days, the reduction in the photocurrent with 1 μg/mL HbA0 binding is increased to 28.7 ± 1.7% and 27.5 ± 1.7%, respectively, which corresponds to a 95.9 ± 5.7% and 92.0 ± 5.6% recovery of the target binding ability. Using the same refolding process, the respective target binding ability of stored HbA1c aptasensors at the 21st and 28th days is 98.2 ± 5.9% and 97.5 ± 5.5% of that of a freshly prepared sensor. The as-prepared aptasensors have a robust b-PM transducer layer, stable covalent linkages and the structurally reversible aptamers so they are sufficiently stable in storage.

### 3.4. Clinical Samples

**Figure 7:**
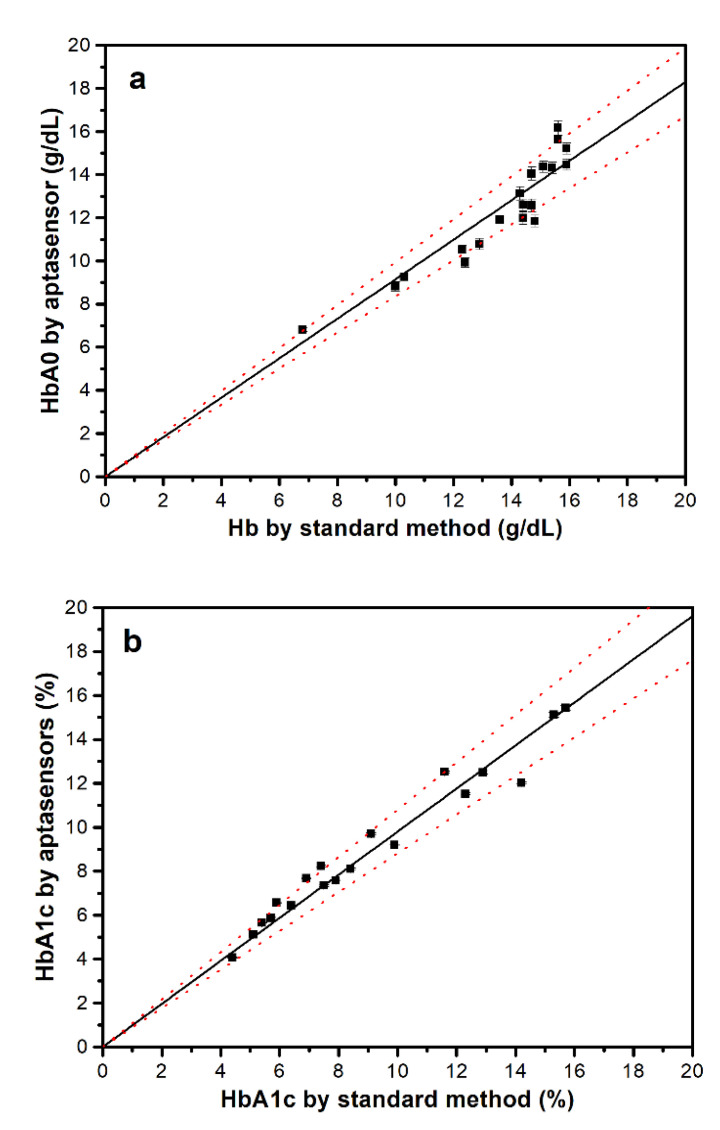
Correlation (**a**) between the aptasensor HbA0 concentrations and the standard measured total Hb concentrations and (**b**) between the HbA1c levels that are measured using the aptasensors and the standard method. The red dotted lines show 90% confidence intervals. All aptasensor data is presented as the average value for three measurements with one standard deviation. The RSDs for the HbA0 and HbA1c concentrations that are determined using their respective aptasensors are, respectively, less than 5% and 6%.

A total of 19 human whole-blood clinical samples were collected by medical professionals and each was analyzed using the developed HbA0 and HbA1 aptasensors to determine their HbA0 and HbA1c concentrations and the HbA1c levels. As shown in Figure 7a, the HbA0 concentrations that are determined using the HbA0 aptasensor correlate well with the total Hb values that are measured using the standard method with a hematology analyzer (correlation gradient = 0.915 ± 0.004; Pearson correlation coefficient, *R* = 0.930). The average ratio of the aptasensor HbA0 concentrations to the standard total Hb concentrations for all of the tested samples is 90.4 ± 6.8%, which agrees well with the average HbA1c levels that are measured using the well-established capillary electrophoresis method (9.05 ± 3.60%).

Figure 7b shows that the HbA1c levels that are determined using the proposed aptasensor technique using Equation (1) also correlate well with the levels that are measured using the capillary electrophoresis method (correlation gradient = 0.981 ± 0.001; Pearson correlation coefficient, *R* = 0.981; inaccuracy = 5.8 ± 4.3%). In comparison with other aptamer-based HbA1c assay methods, the developed photoelectric aptasensors allow direct and label-free detection with the widest HbA1c-level detection range and comparable detection accuracy, sensitivity, specificity, speed and stability (Table S3), so they are suited to clinical applications.

## 4. Conclusions

A BR-containing b-PM monolayer is demonstrated as a potent sensor transducer for human HbA0 and HbA1c detection and as a convenient and reliable tool to determine HbA1c-levels. Antibodies and aptamers are reproducibly immobilized on the b-PM transducer to recognize their respective target Hb molecules, absorbing part of the incident light and consequently causing a decrease in the photocurrent in the sensor. The reproducible and stable HbA0 and HbA1c aptasensors selectively and sensitively recognize their respective target molecules without cross reactivity or interference from common interferents.

An analysis of the human whole-blood clinical samples demonstrates that HbA0 concentrations and HbA1c levels are readily single-step determined in 15 min with satisfactory accuracy. Only 5 μL of 1000- and 5000-fold diluted blood samples is required for the HbA1c and HbA0 aptasensor assays. Both aptasensor chips have a small dimension (length × width × thickness: 2.5 cm × 0.7 cm × 0.1 cm), light weight (0.4 g), and a small sample coating area (diameter: 3 mm). Since the coating amount is little, the material cost to prepare each aptasensor is estimated to be approximately just 0.7 US dollar, including ITO glass, PM, aptamers, and linker reagents. Two of each kind of aptasensor are required to determine a HbA1c level, one for the sample and the other one for the blank binding buffer, so the material cost for each HbA1c determination is less than 3 USD. The current amplifier and oscilloscope can be combined as a small compact unit, so the whole photocurrent measurement setup can be easily miniaturized to become a portable device if the laser unit and the cuvette cell are further replaced with a LED light and a 3D printed module, respectively. The proposed BR-based aptasensor technique allows direct, label-free, fast and simple assay of human HbA1c for both clinical and point-of-care applications.

## Supplementary Materials

The following are available online at https://www.mdpi.com/1424-8220/20/24/7274/s1, Figure S1: Adsorption isotherms, Figure S2: Immunofluorescence analysis for a HbA0-coated HbA0 aptasensor, Figure S3: De-convoluted Raman spectra for various coated ITO electrodes, Figure S4: Cross reactivity between HbA0 and HbA1c aptasensors, Figure S5: AFM topographic images of the b-PM layer on mica after storage in 10 mM PB containing 100 mM NaCl at pH 8.0 and 4 °C for different days, Figure S6: Relative total photocurrent density of various chips after storage for different days, Table S1: Raman-band (cm^−1^) assignment for ITO electrodes that are fabricated with different top layers, Table S2: Decrease in photocurrent for HbA0 and HbA1c aptasensors after incubation with various substances at the specified concentrations, Table S3: Comparison of various aptamer-based HbA1c assay methods.
