# Supplementary material for: Direct and Label-Free Determination of Human Glycated Hemoglobin Levels Using Bacteriorhodopsin as the Biosensor Transducer
Source: Sensors (Basel). 2020 Dec 18;20(24):7274. doi: 10.3390/s20247274 (PMC7765918; doi:10.3390/s20247274)
Supplement: Supplementary file 1 [file sensors-20-07274-s001.pdf]

Supplementary Materials

Direct and label-free determination of human glycated hemoglobin levels using bacteriorhodopsin as the biosensor transducer

Ying-Chin Lin[1,2,3,a], Ching-Yu Lin[4,a], Hsiu-Mei Chen[5,*], Li-Pin Kuo[5], Cheng-En Hsieh[5], Xiang-He Wang[5], Chih-Wen Cheng[5], Chih-Yin Wu[3], Yi-Su Chen[3]

[1] Department of Family Medicine, School of Medicine, College of Medicine, Taipei Medical University, Taipei, 110, Taiwan

[2] Department of Geriatric Medicine, School of Medicine, College of Medicine, Taipei Medical University, Taipei, 110, Taiwan

[3] Department of Family Medicine, Wan Fang Hospital, Taipei Medical University, Taipei, 116, Taiwan

[4] School of Medical Laboratory Science and Biotechnology, College of Medical Science and Technology, Taipei Medical University, Taipei 11031, Taiwan

[5] Department of Chemical Engineering, National Taiwan University of Science and Technology, Taipei 10607, Taiwan

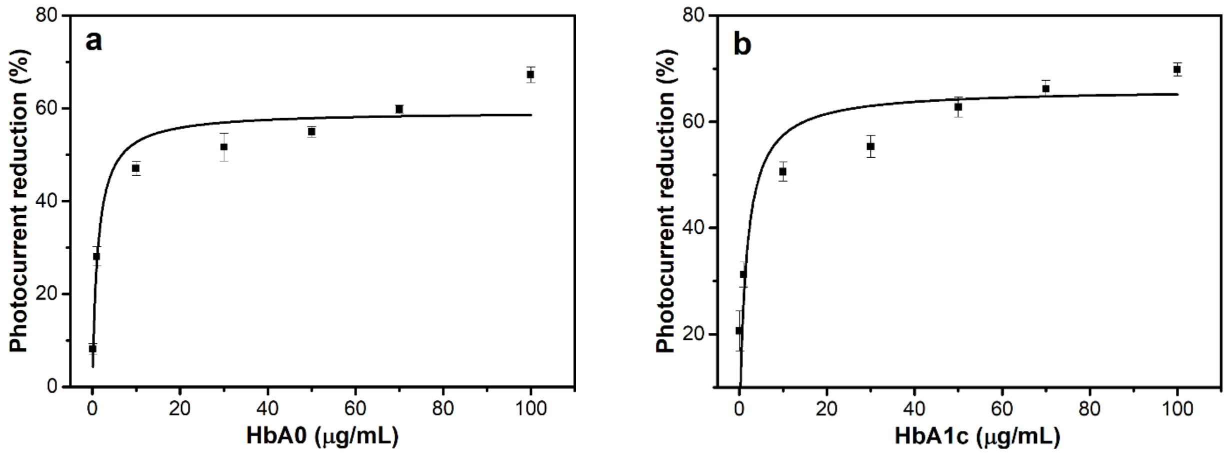

Fig. S1. Adsorption isotherms for (a) HbA0 and (b) HbA1c aptasensors toward pure HbA0 and HbA1c solutions, respectively. The data is from Figs. 2b and 2c, respectively, and is nonlinearly fitted using the Langmuir adsorption model. All data is presented as the average value for three chips of a single type with one standard deviation. The monomeric dissociation constants (K_d) for isotherms (a) and (b) are calculated to be 79 ± 36 nM and 84 ± 43 nM, respectively, using monomeric molecular weights of HbA0 and HbA1c of 15.9 and 18 kDa, respectively.

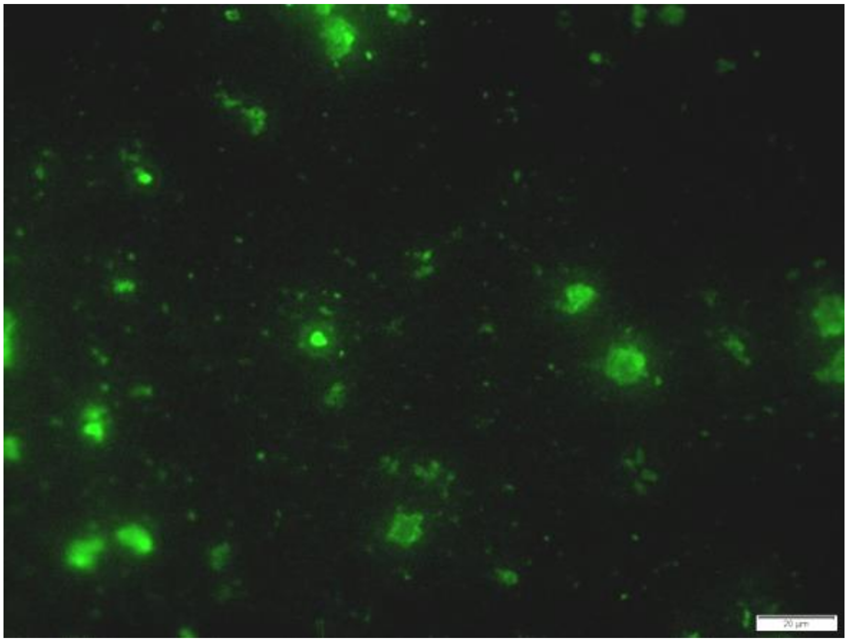

Fig. S2 Immunofluorescence analysis for a HbA0-coated HbA0 aptasensor. The HbA0 aptasensor was drop-coated with 100 μg/mL HbA0, immunolabeled with primary rabbit anti-human Hb antibodies and stained with secondary anti-Rabbit IgG antibodies that were conjugated with Alexa Fluor® 488. The stained HbA0 molecules were visualized using an Olympus IX73 inverted fluorescence microscope. No green spot is present for the aptasensor that is coated with the blank binding buffer.

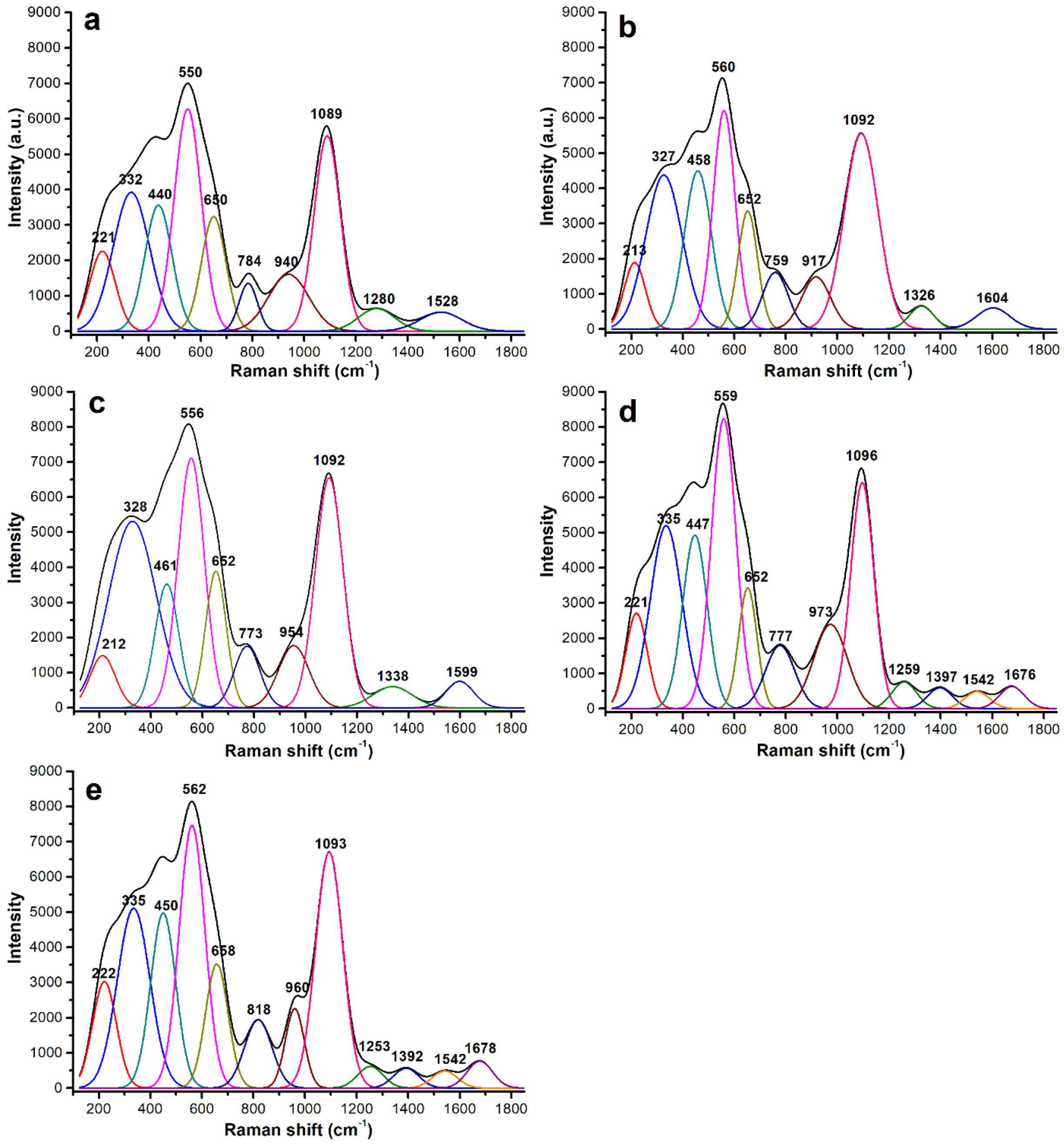

Fig. S3. De-convoluted Raman spectra for ITO electrodes that are fabricated with (a) b-PM, (b) HbA0 aptamers, (c) HbA1c aptamers, (d) HbA0 and (e) HbA1c at the top. A PeakFit deconvolution program was used to identify the bands in each spectrum.

Table S1. Raman-band (cm^{-1}) assignment for ITO electrodes that are fabricated with different top layers[a]

b-PM	HbA0 aptamer	HbA1c aptamer	HbA0	HbA1c	Band assignment	References
221	213	212	221	222	ITO	-
332	327	328	335	335	ITO	-
440	458	461	447	450	ITO	-
550	560	556	559	562	ITO	-
650	652	652	652	658	C-S stretches/Tyr	[S1,S2]
784	759	773	777		Trp	[S1-S3]
				818	HbA1c	[S4]
940	917	954			ITO	-
			973	960	Hb/HbA1c	[S5]
1089	1092	1092	1096	1093	ITO	-
			1259	1253	Hb/HbA1c	[S5]
1280					C-C (C=C) stretching, C-C-H in-plane bends, retinal	[S6,S7]
	1326	1338			DNA bases	[S8,S9]
			1397	1392	Hb/HbA1c	[S4]
1528			1542	1542	C=C stretching, heme/retinal	[S7]
	1604	1599			DNA bases	[S8,S9]
			1676	1678	Hb/HbA1c	[S4]

[a] Data is from the de-convoluted Raman spectra in Fig. S3.

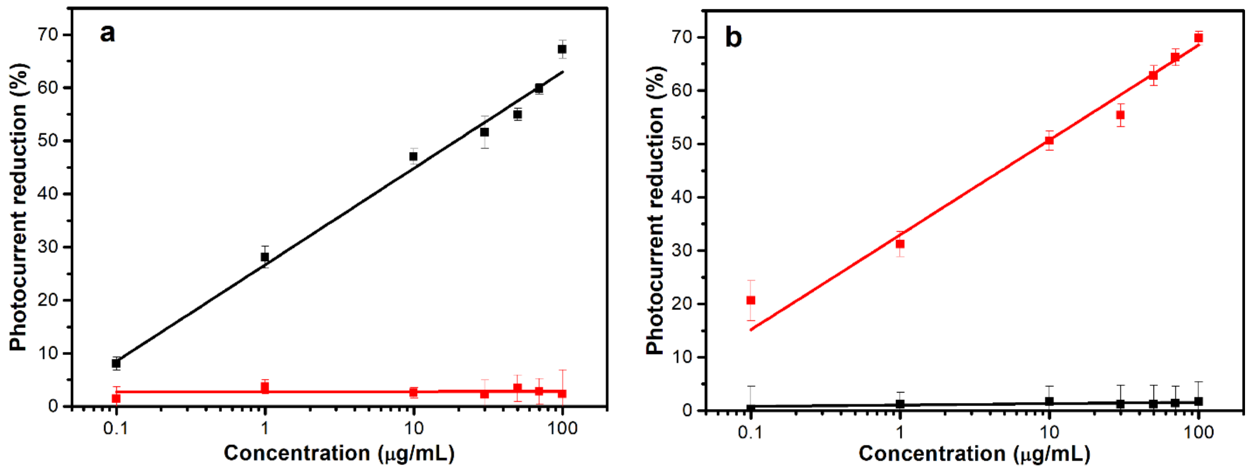

Fig. S4. Cross reactivity between HbA0 and HbA1c aptasensors. Decrease in photocurrent for (a) HbA0 and (b) HbA1c aptasensors on the detection of 0.1-100 μg/mL pure (black) HbA0 and (red) HbA1c solutions, respectively. All data is presented as the average value for three chips of a single type with one standard deviation (RSD < 5%). The average decrease in the photocurrent for the HbA0 aptasensor after incubation with different concentrations of HbA1c is 2.66 ± 0.77% and that for the HbA1c aptasensor with different concentrations of HbA0 is 1.23 ± 0.48%.

Table S2. Decrease in photocurrent for HbA0 and HbA1c aptasensors after incubation with various substances at the specified concentrations[a]

Substance	Concentration	Photocurrent reduction (%)	
		HbA0 aptasensor	HbA1c aptasensor
acetaminophen	80 μg/mL	1.95 ± 1.33	2.93 ± 1.35
acetylsalicylic acid	10 μg/mL	3.90 ± 3.77	1.62 ± 1.87
ascorbic acid	50 μg/mL	3.04 ± 2.81	2.49 ± 1.07
bilirubin	0.2 mg/mL	4.34 ± 3.23	0.75 ± 1.63
glyburide	0.24 μg/mL	2.60 ± 1.86	2.06 ± 4.55
HbA0	5 mg/mL	-	2.27 ± 1.63
HbA1c	0.5 mg/mL	2.61 ± 5.45	-
ibuprofen	0.12 mg/mL	2.82 ± 2.21	0.09 ± 3.86
metformin	25 μg/mL	4.12 ± 3.54	2.69 ± 2.93
triglyceride	30 mg/mL	3.90 ± 4.77	2.28 ± 5.89

[a] Data is presented as the average value for three chips of a single type with one standard deviation.

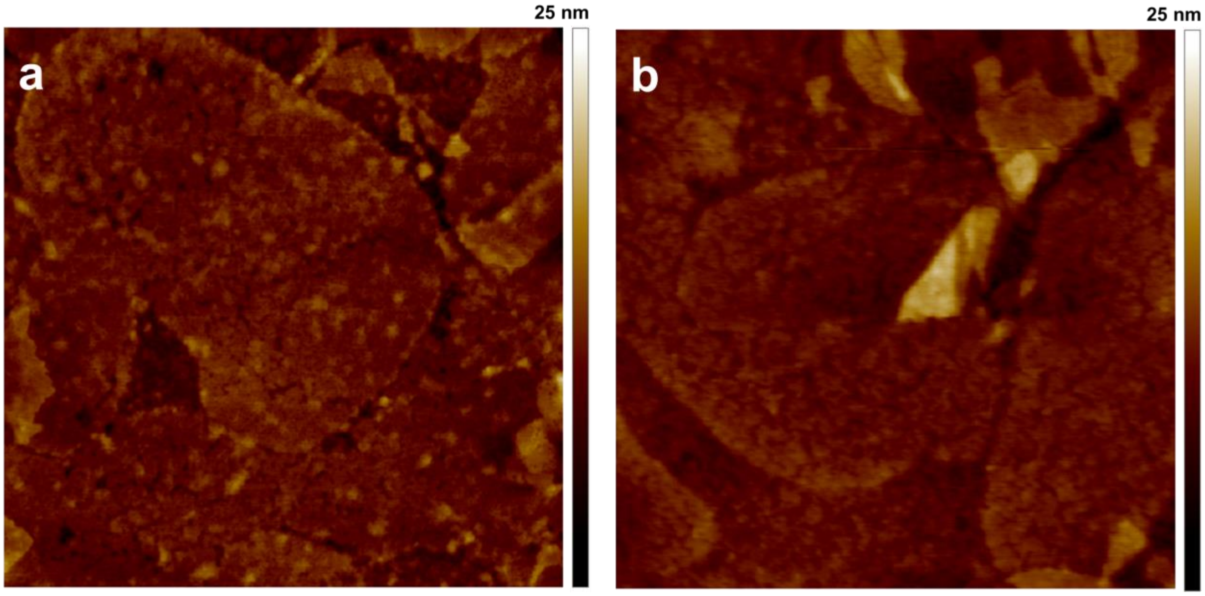

Fig. S5. AFM topographic images of the b-PM layer on mica after storage in 10 mM PB containing 100 mM NaCl at pH 8.0 and 4 °C for (a) 14 and (b) 28 days; scan size: 2 μm

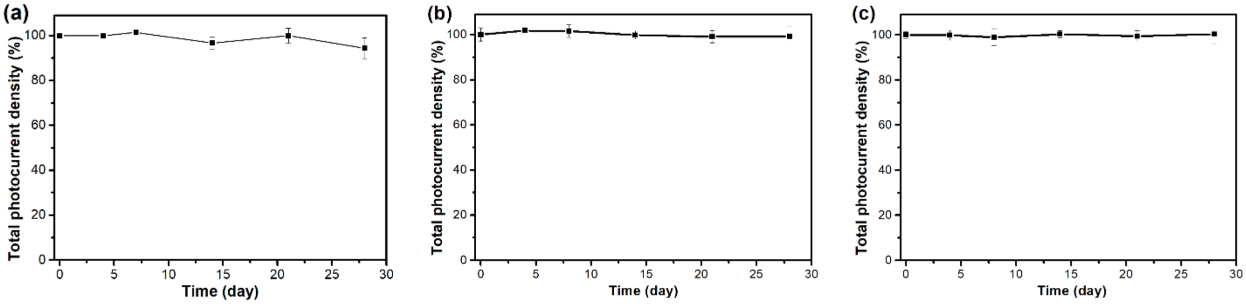

Fig. S6. Relative total photocurrent density for the (a) b-PM chip, (b) HbA0 aptasensor and (c) HbA1c aptasensor after storage in 10 mM PB containing 100 mM NaCl at pH 8.0 and 4 °C for 0-28 days. The values for the total photocurrent density generated by fresh chips of each type are taken as 100%. All data is presented as the average value for three chips of a single type with one standard deviation (RSD < 5%). The average relative total photocurrent density for the HbA0 and HbA1c aptasensors on the 28th day are 99.2 ± 4.5% and 100.3 ± 4.2%, respectively.

Table S3. Comparison of various aptamer-based HbA1c assay methods.

Assay	BR-based aptasensor[a]	Electrochemical sensor	Integrated microfluidic system based on aptamer-antibody sandwich assay via magnetic beads	Microfluidic amperometric dual-sensor	On-chip aptamer-based sandwich assay
Detection method	Photoelectric (electrode: aptamer/ b-PM/GO-OA/APPA /ITO[a])	Electrochemical (electrode: aptamer/AuNPs/SPCE[a])	chemiluminescent	Electrochemical (electrodes: TBO/pTBA @MWCNT/SPCE and aptamer/TBO/pTBA @MWCNT/SPCE[a])	Fluorescent
Direct assay	yes	yes	no	yes	no
Label-free assay	yes	yes	no	yes	no
Detection limit	HbA0: 0.1 μg/mL<br>HbA1c: < 0.1 μg/mL	Total Hb: 0.34 ng/mL<br>HbA1c: 0.20 ng/mL	Total Hb: 8.8 g/dL<br>HbA1c: 0.65 g/dL	Total Hb: 82 nM<br>(~ 5.2 μg/mL)<br>HbA1c: 3.7 nM<br>(~ 0.27 μg/mL)	n.a.[b]
Dynamic range	Both HbA0 and HbA1c: 0.1-100 μg/mL	Both total Hb and HbA1c: 0.1-100 ng/mL	Total Hb: 3.7-14.9 g/dL<br>HbA1c: 0.46-1.85 g/dL	Total Hb: 0.1-10 μM<br>HbA1c: 0.006-0.74 μM	Total Hb:10.8-14.8 g/dL<br>HbA1c: 0.7-2.1 g/dL
Assay/binding time	15-min binding	30-min binding	25-min assay	4-min binding	30-min assay
Blood HbA1c level	4.4-15.7%	6.67-10.47%	4.8-13.2%	5.3-11.8%	4.3-14.0%
Blood amount	< 1 μL blood;<br>5×10^3-fold dilution for HbA0 and 10^3-fold dilution for HbA1c;<br>5 μL dilute solution for each sensor	1 μL blood;<br>10^3 to 10^4-fold dilution for total Hb and 10^2 to 10^5-fold dilution for HbA1c;<br>2 μL dilute solution for each sensor	1 μL blood;<br>49 μL of magnetic bead to mix with the blood	1 μL blood;<br>400-fold dilution	1 μL blood;<br>49 μL of magnetic bead to mix with the blood
Inaccuracy	Average 5.76% (n=19)	n.a.[b]	Average 4.8% (n=4)	*t* value = 0.16<br>(95% confidence level, n=20)	< 7% for HbA1c less than 10 and < 13% for HbA1c higher than 10
Interference	< 4.4% with 9 common interferents (Table S2)	low with BSA[a]	n.a.[b]	Low with acetaminophen, ascorbic acid, dopamine, uric acid, glucose	n.a.[b]
Stability	> 95% activity after 28 days	Few weeks	n.a.[b]	> 95% activity after 50 days	n.a.[b]
Reference	This work	[S10]	[S11]	[S12]	[S13]

[a] APPA: aminopropylphosphonic acid; AuNPs: gold nanoparticles; b-PM: biotinylated purple membrane; BR: bacteriorhodopsin; BSA: bovine serum albumin; GO-OA: graphene oxide-oxidized avidin complex; pTBA@MWCNT: poly(2,2´:5´,5″-terthiophene-3´-p-benzoic acid) and a multi-wall carbon nanotube (MWCNT) composite layer; ITO: indium tin oxide; SPCE: screen printed carbon electrode; TBO: toluidine blue O.

[b] Not available.

References

S1. Painter, P.C.; Koenig, J.L. Raman spectroscopic study of the structure of antibodies. *Biopolymers* **1975**, *14,* 457–468.

S2. Rygula, A.; Majzner, K.; Marzec, K.M.; Kaczor, A.; Pilarczyk, M.; Baranska, M. Raman spectroscopy of proteins: a review. *J. Raman Spectrosc*. **2013**, *44*, 1061–1076.

S3. Fagnano, C.; Fini, G.; Torreggiani, A. Raman spectroscopic study of the avidin-biotin complex. *J. Raman Spectrosc*. **1995**, *26,* 991–995.

S4. Kiran, M.S.; Itoh, T.; Yoshida, K.; Kawashima, N.; Biju, V.; Ishikawa, M. Selective detection of HbA1c using surface enhanced resonance Raman spectroscopy. *Anal. Chem*. **2010**, *82*, 1342–1348.

S5. Barman, I.; Dingari, N.C.; Kang, J.W.; Horowitz, G.L.; Dasari, R.R.; Feld, M.S. Raman spectroscopy-based sensitive and specific detection of glycated hemoglobin. *Anal Chem*. **2012**, *84*, 2474–2482.

S6. Stockburger, M.; Klusmann, W.; Gattermann, H.; Massig, G.; Peters, R. Photochemical cycle of bacteriorhodopsin studied by resonance Raman spectroscopy. *Biochemistry* **1979**, *18*, 4886–4900.

S7. Braiman, M.; Mathies, R. Resonance Raman spectra of bacteriorhodopsin's primary photoproduct: evidence for a distorted 13-cis retinal chromophore. *Proc. Natl. Acad. Sci. USA* **1982**, *79*, 403–407.

S8. De Gelder, J.; De Gussem, K.; Vandenabeele, P.; Moens, L. Reference database of Raman spectra of biological molecules. *J. Raman Spectrosc*. **2007**, *38*, 1133–1147.

S9. Coluccio, M.L.; Gentile, F.; Das, G.; Perozziello, G.; Malara, N.; Alrasheed, S.; Candelor

o, P.; Di Fabrizio, E. From nucleotides to DNA analysis by a SERS substrate of a self similar chain of silver nanospheres. *J. Opt*. **2015**, *17*, 114021.

S10. Eissa, S.; Zourob, M. Aptamer-based label-free electrochemical biosensor array for the detection of total and glycated hemoglobin inhuman whole blood. *Sci. Rep*. **2017**, *7*, 1016.

S11. Chang, K.W.; Li, J.; Yang, C.H.; Shiesh, S.C.; Lee, G.B. An integrated microfluidic system for measurement of glycated hemoglobin levels by using an aptamer-antibody assay on magnetic beads. *Biosens. Bioelectron.* **2015**. *68*, 397–403.

S12. Moon, J.M.; Kim, D.M.; Kim, M.H.; Han, J.Y.; Jung, D.K.; Shim, Y.B. A disposable amperometric dual-sensor for the detection of hemoglobin and glycated hemoglobin in a finger prick blood sample. *Biosens. Bioelectron*. **2017**, *91*, 128–135.

S13. Li, J.; Chang, K.W.; Wang, C.H.; Yang, C.H.; Shiesh, S.C.; Lee, G.B. On-chip, aptamer-based sandwich assay for detection of glycated hemoglobins via magnetic beads. *Biosens. Bioelectron*. **2016**, *79*, 887–893.
